# The effects of individual and area-level socioeconomic status on mortality from cancer of the head and neck in Belgium, 2001-2011

**DOI:** 10.1186/2049-3258-73-S1-P2

**Published:** 2015-09-17

**Authors:** Paulien Hagedoorn, Hadewijch Vandenheede, Katrien Vanthomme, Sylvie Gadeyne

**Affiliations:** 1 Vrije Universiteit Brussel, Brussels, Belgium

## 

Multiple studies observed an independent effect of area characteristics on cancer mortality. Few focused on specific cancer types, although this might yield more information on cancer etiology, risk factors, and at-risk populations. Some research has been conducted on head and neck cancer, observing higher incidence and lower survival from head and neck cancer in deprived areas. Yet, the effect of area characteristics on (mortality from) head and neck cancer remains understudied. This study aims to determine the spatial pattern of head and neck cancer mortality in Belgium, to assess the effect of individual and area-level socioeconomic status on mortality from head and neck cancer, and to estimate the interaction between individual and area-level characteristics. Data are collected from a unique dataset linking census and register data on all Belgian inhabitants aged 40 and above from 2001-2011. Head and neck cancer mortality is defined according to the ICD-10 codes C00-C14 and C30-C32. The (indirectly) standardized mortality ratio (SMR) is calculated by sub-district using the age and sex-specific rates of Belgium as the standard. Multilevel Poisson models are used to estimate the (interaction) effects of individual and area-level socioeconomic status on head and neck cancer, and their contribution to regional variations. Analyses are stratified by age category (40-64; 65+) and by subtype with different etiologies and risk factors. Preliminary results show distinct spatial patterns in the SMR for cancer of the head and neck. A negative association with individual socioeconomic status is observed. The spatial pattern of housing quality and unemployment rate shows similarities to that of head and neck cancer mortality, indicating these area-level characteristics might be associated to mortality of head and neck cancer. The spatial pattern of head and neck cancer also closely resembles that of alcohol-related mortality, suggesting alcohol consumption might be a possible risk factor as well.

